# Public Knowledge and Attitude Regarding Symptoms of Acute Coronary Syndrome and Its Related Risk Factors in Western Region, Saudi Arabia

**DOI:** 10.7759/cureus.63001

**Published:** 2024-06-23

**Authors:** Safa H Alkalash, Ali H Alfaqih, Ebrahem R Alsohabi, Alhassan H Al-Faqih, Ahmed A AL-Hayli, Khalid H Almahmudi, Nawaf H Alrufaydi, Omar S Qandus, Fawaz O Alzamil

**Affiliations:** 1 Community Medicine and Health Care, Umm Al-Qura University, Al-Qunfudah, SAU; 2 Family Medicine, Menoufia University, Shebin Elkom, EGY; 3 Medicine and Surgery, Umm Al-Qura University, Al-Qunfudah, SAU; 4 Medicine, Umm Al-Qura University, Al-Qunfudah, SAU; 5 Family Medicine, Hail Health Cluster, Al-Qunfudah, SAU; 6 Emergency, Al-Qunfudah General Hospital, Al-Qunfudah, SAU; 7 Emergency, King Fahad General Hospital, Jeddah, SAU

**Keywords:** chest pain, symptoms, attitude, knowledge, acute coronary syndrome

## Abstract

Background: Acute coronary syndrome (ACS) is the leading cause of mortality and morbidity worldwide. Recognition of its symptoms affects the time-sensitive benefits of reperfusion therapy. Furthermore, lowering the risk factors can prevent long-term complications.

Objectives: To evaluate the public knowledge and perception of the symptoms and risk factors of ACS in the Saudi-Western region.

Method: A cross-sectional study was conducted on a convenience sample of 733 among the general population in the western region of Saudi Arabia by using the Acute Coronary Syndrome Response Index, with additional questions about risk factors for heart attack and physical activities. The research information was acquired through a self-administered questionnaire without any identifying personal information.

Result: Participants demonstrated awareness of certain ACS symptoms and risk factors. Chest pain was widely recognized (49.2%, n = 361), followed by shortness of breath (44.8%, n = 329), arm pain or shoulder pain (38.6%, n = 283), palpitation (37.3%, n = 274), and fatigue (22.2%, n = 163). A total of 544 (74.2%) and 474 (64.6%) respondents were aware that smoking and obesity are the most common risk factors for ACS, respectively. However, gaps persisted, particularly regarding the association between diabetes mellitus and ACS, with 31.6% (n = 232) reporting diabetes mellitus. A total of 331 (45.2%) and 322 (43.9%) study sample were unsure whether they could identify ACS in themselves or other people. However, 391 (53.3%) decided that they should go to the hospital as soon as possible when they have chest pain that does not stop after 15 minutes. Notably, female participants demonstrated substantially higher knowledge (OR = 2.40, p = 0.001). The study highlights the influence of gender, age, and education on ACS-related awareness.

Conclusion: This study provides valuable insights into ACS awareness in the western region of Saudi Arabia. Relatively older respondents, female participants, and those with postgraduate education were more knowledgeable about ACS than the others. These findings emphasize the importance of tailored interventions for specific demographic groups in enhancing public health outcomes.

## Introduction

The discovery of exorbitant natural resources like oil and gas boosted the economies of Gulf countries, including the Kingdom of Saudi Arabia (KSA) [1], led to massive lifestyle shifts, and heightened the risk of cardiovascular disorders (CVDs) and related diseases among the Arab population [2-3]. Acute coronary syndrome (ACS) describes individuals who may be suffering from acute myocardial ischemia or infarction due to a significant decrease in coronary blood flow. Unstable angina (UA), non-ST-elevation myocardial infarction (NSTEMI), and ST-elevation myocardial infarction (STEMI) are the three types of ACS [4]. Chest pains, repeated discomfort in the arm (left) or jaw angle, pressure, and sweating are frequent and recognizable ACS symptoms [5]. However, a number of non-specific symptoms of ACS, such as heartburn, vomiting, or nausea, and epigastric discomfort, are not recognized by patients [5]. The patient is often reluctant to seek prompt medical attention due to these vague symptoms, which are typically misinterpreted as non-cardiac in nature [6]. The definitive diagnosis of ACS is made using cardiac biomarkers and electrocardiographic variations; however, patient awareness of ACS within the golden period is crucial and affects the disease prognosis [7-8]. Thrombolytics, percutaneous interventions of the coronary pathway, and instant reperfusion help deal with ACS [7].

Globally, acute coronary syndrome (ACS) is the leading cause of mortality and morbidity [9]. An estimated 17.9 million deaths worldwide in 2019 were attributed to CVDs, accounting for 32% of all deaths. Eighty-five percent of these fatalities were brought on by heart attacks and strokes. Three-quarters of the 17 million premature deaths (dead before age 70) attributed to noncommunicable illnesses in 2019 (CVDs) were caused by these disorders [10]. Western enculturation and urbanization have substantially contributed to exalting the ACS rates [11].

There has been a notable transformation in Saudi Arabia, where the adoption of a Western lifestyle is linked to an increase in the prevalence of cardiovascular disease risk factors [12]. The ACS incidence rate is 8.2% in Saudi Arabia [13]. New research from Saudi Arabia revealed that patients with ACS were more likely to be male (67.4%) and urban (75.8%). In addition, 33.4% of NSTEMI patients had grade 1 dyspnea (51.7%); however, patients with STEMI had a higher risk for mortality [14]. In terms of the management of acute cardiac ischemia, once intervention therapy is started during the first seventy minutes, the mortality decrease approaches half [15]. According to a prior study conducted in Saudi Arabia, the median pre-hospital delay for men was five hours, and for women, it was 12.9 hours [16]. Individuals' understanding of their health and illness is the key means of detecting their health state and serving as a signal to seek medical attention. Therefore, the aim of this study was to investigate the general knowledge and attitudes toward ACS symptoms in the Saudi-Western region.

## Materials and methods

Study design

An observational cross-sectional study included 733 participants.

Study period

The research data were collected over five months, from February to June 2023.

Study setting

The western region of Saudi Arabia was the study setting. It is split into 26 governorates between its two principal provinces: Makkah, which includes 17 governorates, and Al-Madinah, which involves nine governorates.

Study population (eligibility criteria)

The study involved Saudi and non-Saudi people of both genders, 18 years of age and older, living in the Saudi-Western region.

Sample size calculation

The sample size was calculated with EPI-INFO (Center for Disease Control and Prevention (CDC); Atlanta, Georgia, USA). It depended on the total population in the Saudi-Western region (8,325,304), the frequency of public knowledge about ACS from a previous study [17], a 95% level of confidence, a 5% margin of error, and a 10% non-response rate. Finally, a total of 733 respondents completed this survey.

Tool and procedure for data collection

A validated questionnaire was adopted from a previous study [18]. It was digitally formatted using Google Forms and distributed through online platforms such as WhatsApp and Telegram to reach a diverse participant pool. The questionnaire used started with a cover page to transparently communicate the study's purpose and secure participants' informed consent. The questionnaire comprised two distinct sections to gather comprehensive information. The initial section focused on socio-demographic variables, encompassing age, gender, nationality, education level, and marital status. The second section incorporated the ACS Response Index developed by Riegel et al. [18], supplemented by additional inquiries primarily addressing participants' attitudes and knowledge regarding risk factors associated with acute coronary syndrome. This meticulous design aimed to capture a holistic understanding of the participants' socio-demographic characteristics and their perspectives on acute coronary syndrome, fostering a comprehensive and nuanced analysis of the survey data. A pilot study was conducted in January 2023 and targeted 45 responses. The questionnaire items were pre-tested to ensure their understandability by the study subjects. No modifications were needed.

Data analysis

The data underwent meticulous processing, beginning with entry into Excel and subsequent exportation to STATA version 17.1 (StataCorp LLC, Texas, USA) for comprehensive statistical analysis. Descriptive statistics, including mean ± SD (mean and standard deviation) for continuous variables and frequency (%) for categorical variables, were employed to provide a comprehensive overview of the dataset. Multiple logistic regression was then employed to ascertain the independent predictors of adequate versus inadequate knowledge, as well as favorable versus unfavorable attitudes. The analysis encompassed a broad spectrum of socio-demographic, clinical characteristics, and psychosocial variables to comprehensively capture the multifaceted determinants influencing knowledge, attitudes, and beliefs related to acute coronary syndrome. A P value less than 0.05 denotes statistical significance.

Ethical consideration

The ethical approval for this research was obtained from the scientific research ethics committee at Umm Al-Qura University before starting data collection (approval no.: HAPO-02-K-012-2023-05-1618). Data collection was done with the goal of ensuring its confidentiality.

## Results

Among 733 valid responses, 68.5% (n = 502) were female. Age-wise, the majority fell within the 31-59 age group (51.5%, n = 378). Most participants were Saudi nationals (90.3%, n = 662), with the largest proportion holding university degrees (65.3%, n = 479). Marital status revealed a diverse range, with single individuals comprising 41.5% (n = 304), married at 50.2% (n = 368), and divorced and widowed participants at 1.5% (n = 11) and 6.8% (n = 50), respectively. Individuals employed in the government sector (23.7%, n = 174), private sector (19.6%, n = 144), and students (26.9%, n = 195). Geographically, the majority resided in the Makkah region and its provinces (69.2%, n = 507). Monthly income exhibited variation, with 46.1% (n = 338) earning between 15,000 and 20,000 Saudi riyals, and medical history data highlighted the prevalence of chronic conditions, with 73.9% (n = 542) reporting no chronic diseases and others indicating diabetes mellitus (7.2%, n = 53) and hypertension (7.6%, n = 56) (Table 1).

**Table 1:** The socio-demographic profile of the study sample in western regions of Saudi Arabia

Variables	Frequency (n = 733)	Percentage (%)
Sex		
Male	231	31.5
Female	502	68.5
Age		
18-30	336	45.8
31- 59	378	51.6
60 and above	19	2.6
Nationality		
Saudi	662	90.3
Non-Saudi	71	9.7
Education level		
Intermediate school and below	180	24.6
Secondary school	66	9.0
University	479	65.3
Postgraduate	8	1.1
Marital status		
Single	304	41.5
Married	368	50.2
Widowed	50	6.8
Divorced	11	1.5
Occupation		
Government sector employee	174	23.7
Private sector employee	144	19.6
Retired	40	5.5
Student	195	26.6
Unemployed	180	24.6
Residence		
Al-Madinah region and its provinces	226	30.8
Makkah region and its provinces	507	69.2
Monthly income		
Less than 5,000	104	14.2
5,000 to less than 10,000	71	9.7
10,000 to less than 15,000	176	24.0
15,000 to less than 20,000	338	46. 1
20,000 or more	44	6.0
Medical history		
No chronic diseases	542	73.9
Diabetes mellitus	53	7.2
Hypertension	56	7.6
Dyslipidemia	25	3.4
Stroke	14	1.9
Ischemic heart disease	17	2.3
Bronchial asthma	19	2.6
Other diseases	14	1.9

The comprehensive analysis of ACS symptoms reported by 733 participants in the study reveals a diverse array of manifestations. Chest-related symptoms, including chest pain, pressure, and tightness, and chest discomfort (heaviness, burning, and tenderness) were predominant, collectively reported by almost half of the participants (49.2%, n = 361 and 34.7%, n = 255, respectively). Additionally, a significant proportion experienced symptoms indicative of cardiovascular distress, such as palpitations or rapid heart rate (37.3%, n = 274) and shortness of breath or difficulty breathing (44.8%, n = 329). Pain in the arms or shoulders (38.6%, n = 283) and numbness or tingling in the arms or hands (31.6%, n = 232) also featured prominently. Symptoms suggestive of systemic distress, including loss of consciousness or fainting (23.4%, n = 172) and weakness or fatigue (22.2%, n = 163), were reported by notable percentages of participants. Less common symptoms included lower abdominal pain (6.4%, n = 47), cough (8.4%, n = 62), and heartburn/indigestion/stomach problems (6.9%, n = 51). Furthermore, participants reported symptoms affecting various regions, such as jaw pain (11.1%, n = 82) and neck pain (8.7%, n = 64). The breadth of reported symptoms provides a nuanced understanding of the diverse ways in which acute coronary syndrome can manifest, emphasizing the importance of recognizing a spectrum of indicators for timely intervention and preventive measures in cardiovascular health (Table 2).

**Table 2:** Knowledge of the symptoms associated with acute coronary syndrome among the study subjects in the Saudi western region

Symptoms	Frequency (n = 733)	Percentage (%)
Chest pain/pressure/tightness	361	49.2
Shortness of breath/difficulty breathing	329	44.8
Arm pain or shoulder pain	283	38.6
Palpitations/rapid heart rate	274	37.3
Chest discomfort (heaviness, burning, tenderness)	255	34.7
Sweating	234	31.9
Numbness/tingling in arm or hand	232	31.6
Loss of consciousness/fainting	172	23.4
Dizziness, lightheadedness	168	22.9
Weakness/fatigue	163	22.2
Slurred speech	117	15.9
Pale, ashen, loss/change of color	109	14.8
Nausea/vomiting	90	12.2
Back pain	86	11.7
Headache	86	11.7
Jaw pain	82	11. 1
Neck pain	64	8.7
Cough	62	8.4
Heartburn/indigestion/stomach problem	51	6.9
Lower abdominal pain	47	6.4

**Figure 1:**
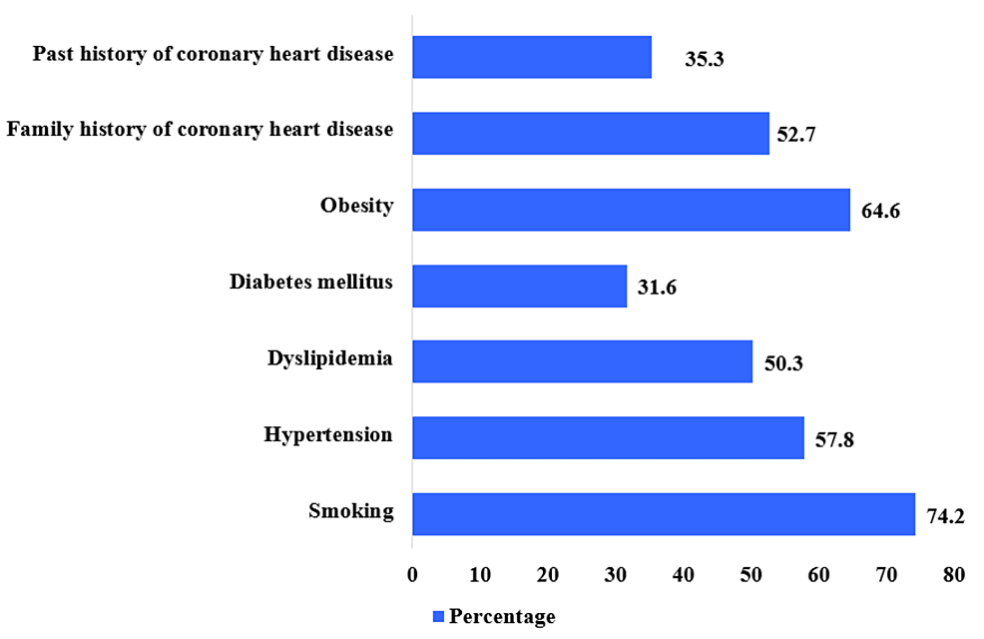
Knowledge of risk factors associated with acute coronary artery disease among individuals in the Saudi western region

Regarding knowledge about risk factors for acute coronary syndrome, smoking emerged as the most prevalent risk factor (74.2%, n = 544), followed by obesity (64.6%, n = 474). Hypertension and high levels of cholesterol were reported by 57.8% (n = 424) and 50.3% (n = 369), respectively. However, diabetes mellitus was known by 31.6% (n = 232) of participants as a risk factor for the development of ACS (Figure 1).

Regarding the participants' attitudes toward recognizing symptoms of coronary artery disease in the Saudi western region. Notably, a significant proportion expressed varying degrees of uncertainty regarding their ability to recognize symptoms, both in themselves and others. For recognizing signs and symptoms in someone else, 30.4% (n = 223) were not at all sure, while 45.2% (n = 331) felt a little sure. Similar patterns were observed for self-recognition, with 27.4% (n = 201) not at all sure and 43.9% (n = 322) a little sure. Participants also exhibited uncertainty in distinguishing heart attack symptoms from other medical problems; 38.2% (n = 280) were not at all sure, and 37.2% (n = 273) were a little sure. Confidence in getting help for a potential heart attack varied, with 34.5% (n = 253) expressing doubt about aiding someone else and 37.5% (n = 275) feeling uncertain about seeking help for themselves (Table 3).

**Table 3:** The participants' attitudes toward recognizing the symptoms of coronary artery disease in the Saudi western region

	Not at all N (%)	Little sure N (%)	Pretty sure N (%)	Very sure N (%)
You could recognize the signs and symptoms of a heart attack in someone else	223 (30.4)	331 (45.2)	128 (17.5)	51 (7.0)
You could recognize the signs and symptoms of a heart attack in yourself	201 (27.4)	322 (43.9)	149 (20.3)	61 (8.3)
You can tell the difference between the signs or symptoms of a heart attack and other medical problems	280 (38.2)	273 (37.2)	128 (17.5)	52 (7.1)
You could get help for someone if you thought they were having a heart attack	253 (34.5)	257 (35.1)	150 (20.5)	73 (10.0)
You could get help for yourself if you thought you were having a heart attack	275 (37.5)	264 (36.0)	122 (16.6)	72 (9.8)

The majority of the study sample strongly agreed (53.3%, n = 391) or agreed (28.9%, n = 212) that immediate hospitalization is crucial when chest pain persists beyond 15 minutes. In contrast, a substantial proportion (42.6%, n = 312) expressed apprehension about potential embarrassment when seeking help for a suspected heart attack. Opinions diverged concerning the necessity of certainty before heading to the hospital, with 22.4% (n = 164) strongly agreeing, 19.6% (n = 144) agreeing, 21.8% (n = 160) disagreeing, and 36.2% (n = 265) strongly disagreeing. Financial considerations come into play, with 22.5% (n = 165) strongly agreeing and 20.2% (n = 148) agreeing that the cost of medical care influences their decision to be sure about a heart attack before seeking hospitalization. In contrast, 17.7% (n = 130) disagreed, and 39.6% (n = 290) strongly disagreed with this notion. Despite the uncertainty, a substantial majority recognized the urgency of seeking medical attention for chest pain, with 39.0% (n = 286) strongly agreeing and 31.1% (n = 228) agreeing, even though they are not very sure it is a heart attack. The inclination to promptly visit the hospital in case of a suspected heart attack is prevalent, with 55.1% (n = 404) strongly agreeing and 21.0% (n = 154) agreeing. However, a notable 10.4% (n = 76) expressed hesitation, and 13.5% (n = 99) strongly disagreed with immediate hospitalization (Table 4).

**Table 4:** The perceptions and beliefs held by participants regarding myocardial infarction (MI)

	Strongly agree N (%)	Agree N (%)	Disagree N (%)	Strongly disagree N (%)
If I have chest pain that does not stop after 15 minutes, I should get to the hospital as soon as possible	391 (53.3)	212 (28.9)	80 (10.9)	50 (6.8)
I would be embarrassed to go to the hospital if I thought I was having a heart attack, but I wasn't	143 (19.5)	143 (19.5)	135 (18.4)	312 (42.6)
If I thought I was having a heart attack, I would wait until I was very sure before going to hospital	164 (22.4)	144 (19.6)	160 (21.8)	265 (36.2)
If I thought I was having a heart attack, I would rather have someone drive me to the hospital than have an ambulance come to my home	202 (27.6)	211 (28.8)	155 (21.1)	165 (22.5)
Because of the cost of medical care, I would want to be sure I was having a heart attack before going to the hospital	165 (22.5)	148 (20.2)	130 (17.7)	290 (39.6)
If I'm having chest pain and I'm not very sure if it is a heart attack, I should go to the hospital	286 (39.0)	228 (31.1)	136 (18.6)	83 (11.3)
If I thought I was having a heart attack, I would go to the hospital right away	404 (55.1)	154 (21.0)	76 (10.4)	99 (13.5)

To identify independent predictors of knowledge levels regarding acute coronary syndrome (ACS), a multiple logistic regression analysis was employed. The predictor variables included in the analysis were gender, age, nationality, education level, and marital status. Notably, gender appears to play a significant role, with female participants demonstrating a substantially higher likelihood of possessing increased knowledge compared to their male counterparts (OR = 2.40, p = 0.001). Age-wise, individuals in the 31-59 age group exhibit a lower odds ratio (OR = 0.62, p = 0.014), while those 60 years of age or older have a higher odds ratio (OR = 1.55, p = 0.003), indicating a potential association with higher knowledge levels compared to other age categories. Moreover, education level emerges as a noteworthy factor, as participants with a postgraduate education exhibit a higher odds ratio (OR = 1.38, p = 0.01) (Table 5).

**Table 5:** Logistic regression to detect factors affecting participants' knowledge levels

Variables	OR (95% CI)	p-value
Sex		
Male	0.93 (0.17-4.97)	0.17
Female	2.40 (0. 16-2. 53)	0.001
Age		
18-30	1.07 (0.08-1.32)	0.76
31-59	0.62 (0.60-1.01)	0.014
60 and above	1.55 (0.14-1.63)	0.003
Nationality		
Saudi	0.47 (0.19-0.52)	0.89
Non-Saudi	1.16 (0.44-1.69)	0.76
Education level		
Intermediate school and below	1.22 (0.98-1.74)	0.61
Secondary school	1.00 (0.62-1.09)	0.51
University	0.85 (0.59-1.37)	0.05
Postgraduate	1.38 (1.22-2.31)	0.01
Marital status		
Married	1.17 (0.86-1.52)	0.45
Single	0.81 (0.24-0.99)	0.71
Divorced	1.21 (1.17-1.28)	0.77
Widowed	0.73 (0.53-1.31)	0.06

## Discussion

Acute coronary syndrome (ACS) is a serious condition that can be fatal if not treated promptly. Despite its diagnosis being done in healthcare facilities through cardiac investigations, public awareness of its symptoms within the golden period is very important to save patients' lives [7,8]. The present study offers a comprehensive overview of the public understanding of acute coronary syndrome in the western regions of Saudi Arabia. The diverse demographic profile reveals a predominantly female and relatively young population, with a notable frequency of university education. The high percentage of participants reporting no chronic diseases suggests a relatively healthy cohort. According to a thorough examination of the participants' knowledge of ACS symptoms, the majority of them are aware that the most typical symptom of ACS is chest pain, which is followed by arm or shoulder pain, shortness of breath, palpitation, and fatigue. However, many people are oblivious to other symptoms, like heartburn, indigestion, and abdominal pain. The current results on the most prevalent ACS symptoms were in line with previous research [17,19-20], maybe as a result of the widespread recognition of chest pain and discomfort as typical ACS symptoms.

In the present study, participants demonstrated a notable awareness of smoking as the most prevalent risk factor for cardiovascular disease, followed by obesity, dyslipidemia, and hypertension. A substantial 31.6% (n = 232) of respondents reported diabetes mellitus as a risk for ACS. These findings align with previous studies, where smoking emerged as the most commonly identified risk factor among the general population [21-22]. Additionally, Alwakeel et al. reported a lack of recognition among patients regarding the association between diabetes mellitus and acute coronary syndrome (ACS) [21], which resonates with the observations in the current study. It is noteworthy that while obesity and smoking are modifiable risk factors, there remains a crucial need to enhance public awareness concerning the risks of ischemic heart disease associated with these factors. Conversely, the present study indicates a limited understanding of significant heart attack risk factors, particularly diabetes. This echoes the findings of numerous prior studies, emphasizing the importance of targeted educational initiatives to address knowledge gaps and promote cardiovascular health awareness [19-22].

This study found that female participants were more knowledgeable about ACS compared to their male counterparts which disagreed with the previous findings of researchers from low and low- and middle-income countries [23-24]. According to this research, women possess greater knowledge, which is corroborated by research done in wealthy nations. Al Harbi et al. and Demisse et al. reported that female patients exhibited a higher level of knowledge regarding acute coronary syndrome (ACS) compared to males [17-25]. Similarly, the recent findings are consistent with published literature suggesting that older participants tend to know more about ACS compared to their younger counterparts [20-21,25]. This phenomenon could be explained by the fact that older individuals are more likely to present with additional risk factors and comorbidities, influencing their understanding of risk in contrast to younger adults, who may perceive themselves as generally healthy. The study results align with existing literature, indicating that individuals with higher levels of education exhibited greater knowledge [19,21] and a heightened perceived risk of acute coronary syndrome (ACS). This significant relationship may be attributed to the likelihood that well-educated individuals have access to ACS-related information from diverse sources compared to those with lower levels of education. These congruent findings underscore the importance of tailoring educational interventions to different demographic groups, considering educational levels and age as key determinants of knowledge, attitudes, beliefs, and risk perceptions related to ACS.

Despite the valuable insights provided by this study, certain limitations must be enumerated. The cross-sectional design limits causal inferences, and the reliance on self-reported data introduces potential biases. Future research should consider longitudinal approaches and incorporate objective clinical measures. Additionally, the study's regional focus may limit generalizability, urging the inclusion of a more diverse sample. Recommendations include expanding the scope of risk factors assessed, tailoring interventions to cultural nuances, and implementing collaborative public health campaigns. By addressing these limitations and embracing these recommendations, future research and initiatives can contribute to a more comprehensive understanding of ACS and improve preventive strategies in the studied population.

## Conclusions

In conclusion, this study highlights that chest pain, arm or shoulder pain, shortness of breath, and palpitations are the most common symptoms of ACS. Smoking, obesity, hypertension, and dyslipidemia are the most known risk factors for the general population. Notable gaps persisted, particularly in recognizing the association between diabetes mellitus and ACS. Female, relatively older, and highly educated participants are more knowledgeable about ACS, which highlighted the need for targeted educational initiatives to address knowledge gaps and promote cardiovascular health awareness. Family physicians and their teams in primary healthcare settings should take the initiative to educate all healthcare visitors about ACS. They should focus on the drawbacks of time delays in seeking healthcare and their impact on patients' lives.
